# I-V and C-V Characterization of a High-Responsivity Graphene/Silicon Photodiode with Embedded MOS Capacitor

**DOI:** 10.3390/nano7070158

**Published:** 2017-06-27

**Authors:** Giuseppe Luongo, Filippo Giubileo, Luca Genovese, Laura Iemmo, Nadia Martucciello, Antonio Di Bartolomeo

**Affiliations:** 1 Dipartimento di Fisica “E. R. Caianiello”, Università di Salerno, via Giovanni Paolo II 132, 84084 Fisciano, Italy; giluongo@unisa.it (G.L.); genoveseluca89@gmail.com (L.G.); liemmo@unisa.it (L.I.); 2 CNR-SPIN Salerno, via Giovanni Paolo II 132, 84084 Fisciano, Italy; filippo.giubileo@spin.cnr.it (F.G.); nadia.martucciello@spin.cnr.it (N.M.)

**Keywords:** graphene, Schottky barrier, MOS capacitor, photodiode, photocurrent

## Abstract

We study the effect of temperature and light on the I-V and C-V characteristics of a graphene/silicon Schottky diode. The device exhibits a reverse-bias photocurrent exceeding the forward current and achieves a photoresponsivity as high as 2.5 A/W. We show that the enhanced photocurrent is due to photo-generated carriers injected in the graphene/Si junction from the parasitic graphene/SiO_2_/Si capacitor connected in parallel to the diode. The same mechanism can occur with thermally generated carriers, which contribute to the high leakage current often observed in graphene/Si junctions.

## 1. Introduction

Graphene is a 2D structure possessing high thermal conductivity [1], high mobility and electrical conductivity [2], maximum surface to volume ratio, low contact resistance [3,4,5] and easy down-scaling [6]. All these properties make it suitable for electronics devices [7], chemical sensors, photodetectors [8] and solar cells [9]. The graphene/silicon (Gr/Si) junction is one of the simplest devices in a hybrid graphene-semiconductor technology. It offers the opportunity to study the physical phenomena that occur at the interface between a gapless 2D and a semiconducting 3D material. The underlying physics in Gr/Si junctions is not yet fully understood. These devices can behave as Schottky diodes and exhibit a rectifying behavior, with current-voltage (I-V) forward characteristics described by the thermionic theory and bias-dependent reverse saturation current. The growing reverse-bias current has been explained by the modulation of the Schottky barrier caused by the low density of states of graphene [10].

**Figure 1:**
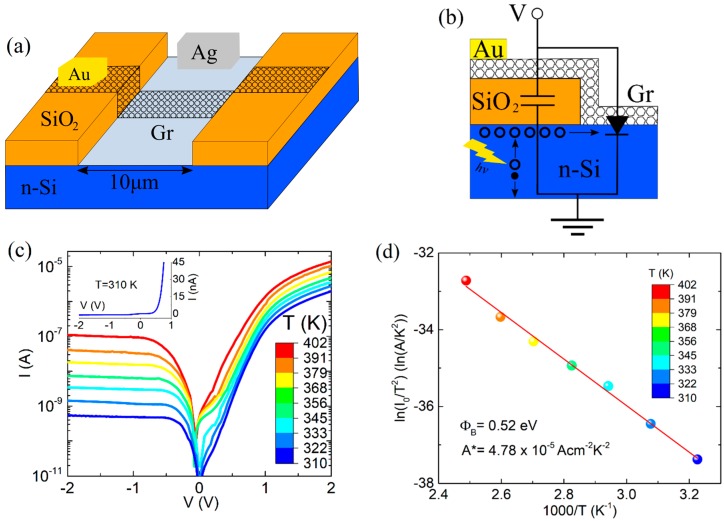
(**a**) Schematic view of the device and (**b**) equivalent circuit; (**c**) I-V characteristic of the Gr/Si junction for decreasing temperature from 400 K to 300 K. The temperature values listed in the figure are measured with an error of ±1 K. The inset shows the I-V characteristic at 310 K in linear scale; (**d**) Richardson plot of $\ln\left(I_{0}/T^{2}\right)$ versus $10^{3}/T$.

In this paper, we characterize a planar Gr/Si junction where part of the graphene is in contact with the n-type silicon, while the remaining part extends over the SiO_2_ insulating layer, forming a metal-oxide-semiconductor (MOS) capacitor (Figure 1a). We demonstrate that the Gr/SiO_2_/Si MOS capacitor (Figure 1b) is able to affect the electrical features of the Gr/Si junction and contributes to its high optical responsivity.

## 2. Results and Discussion

Figure 1c shows the I-V characteristics of the device in semi-logarithmic scale, measured in dark and in the 300−400 K temperature range. The device exhibits a rectifying behavior, with a rectification ratio of up to $10^{3}$, saturated reverse current, turn-on voltage of ~0.5 V at 1 nA current and 310 K, and forward current exponentially growing until reaching the series-resistance $R_{S}$ limited region, characterized by a downward curvature.

The I-V behavior can be expressed by the thermionic emission model [10]:

(1) $$I=I_{0}\left[\exp\left(\frac{qV}{nkT}\right)-1\right]$$

where n is the ideality factor, q is the electron charge, T the temperature, and k the Boltzmann constant. $I_{0}$ is the reverse saturation current (leakage) which is expressed as

(2) $$I_{0}=AA^{*}T^{2}\exp\left(-\frac{\Phi_{b0}}{kT}\right)$$

with $A=4\times 10^{-4}{\;\mathrm{cm}}^{2}$ the active device area, $A^{*}=112\;A{\mathrm{cm}}^{-2}K^{-2}$ the theoretical effective Richardson constant for n-Si and $\Phi_{b0}$ the Schottky barrier height. The ideality factor accounts for mechanisms other than pure thermionic injection as well as for defects or unwanted insulating layers, inadvertently introduced at the Gr/Si interface during the fabrication process. Defects and insulating patches can introduce Schottky barrier inhomogeneity.

We extracted the Schottky barrier height and the effective Richardson constant from the Richardson plot shown in Figure 1d. Rewriting Equation (2) in the form:

(3) $$\ln\left(\frac{I_{0}}{T^{2}}\right)=\ln\left(AA^{*}\right)-\frac{\Phi_{b0}}{k}\frac{1}{T}$$

$\Phi_{b0}$ and $A^{*}$ can be evaluated from the slope and the intercept of the plot of $\ln\left(I_{0}⁄T^{2}\right)$ vs. $10^{3}/T$, respectively. The resulting $A^{*}=4.78\times 10^{-5}\;A{\mathrm{cm}}^{-2}{\;K}^{-2}$ is significantly lower than the theoretical value in metal-semiconductor junctions, and $\Phi_{b0}=0.52\;\mathrm{eV}$. The origin of a lower effective Richardson constant, which has often been a matter of discussion [11,12], can be attributed to the presence of an insulating layer at Gr/Si interface, such as native oxide. Rewriting Equation (2) as:

(4) $$I_{0}=AA^{*}T^{2}\exp\left(-\chi^{1/2}\delta \right)\exp\left(-\frac{\Phi_{b0}}{kT}\right)$$

with the introduction of a tunneling attenuation factor exp(−χ12 δ), where χ (expressed in eV) is the mean barrier height and δ (expressed in Å) is the thickness of the insulating layer (a dimensional constant of ${\left[2\left(2m^{*}\right)\hbar^{2}\right]}^{\frac{1}{2}}\approx 1.01{\;\mathrm{eV}}^{\frac{1}{2}}\;{Å}^{-1}$ is commonly omitted), $m^{*}$ is the effective electron mass, ℏ is the reduced Planck constant, the Richardson constant can be redefined as

(5) $$A^{**}=A^{*}\exp\left(-\chi^{1/2}\delta \right)$$

Assuming that $A^{*}=112\;A{\mathrm{cm}}^{-2}\;K^{-2}$ and χ≈3 eV for a SiO_2_ interfacial layer, $A^{**}=4.78\times 10^{-5}\;A{\mathrm{cm}}^{-2}{\;K}^{-2}$ and δ∼8.5 Å are extracted from the intercept of the Richardson plot. The interface insulating layer, which has been confirmed by X-ray photoelectron spectroscopy measurements (not reported, here, for brevity), reduces the minority-carrier tunneling current without affecting the majority carrier current, which is from diffusion, and raises the majority injection efficiency [13,14].

The series resistance $R_{S}$ can be evaluated using Cheung’s method [15]. Taking into account the voltage drop on the series resistance, $R_{S}I$, Equation (1) becomes

(6) $$I=I_{0}\left[\exp\left(\frac{q\left(V-R_{S}I\right)}{nkT}\right)-1\right]$$

From Equation (6), when −1 can be neglected, two equations can be derived, both yielding $R_{S}$:

(7) $$\frac{dV}{d\ln\left(I\right)}=IR_{S}+n\left(\frac{kT}{q}\right)$$

and

(8) $$H\left(I\right)=V-n\left(\frac{kT}{q}\right)\ln\left(\frac{I}{AA^{**}T^{2}}\right)$$

where H(I) is defined as

(9) $$H\left(I\right)=IR_{S}+n\Phi_{b0}$$

**Figure 2:**
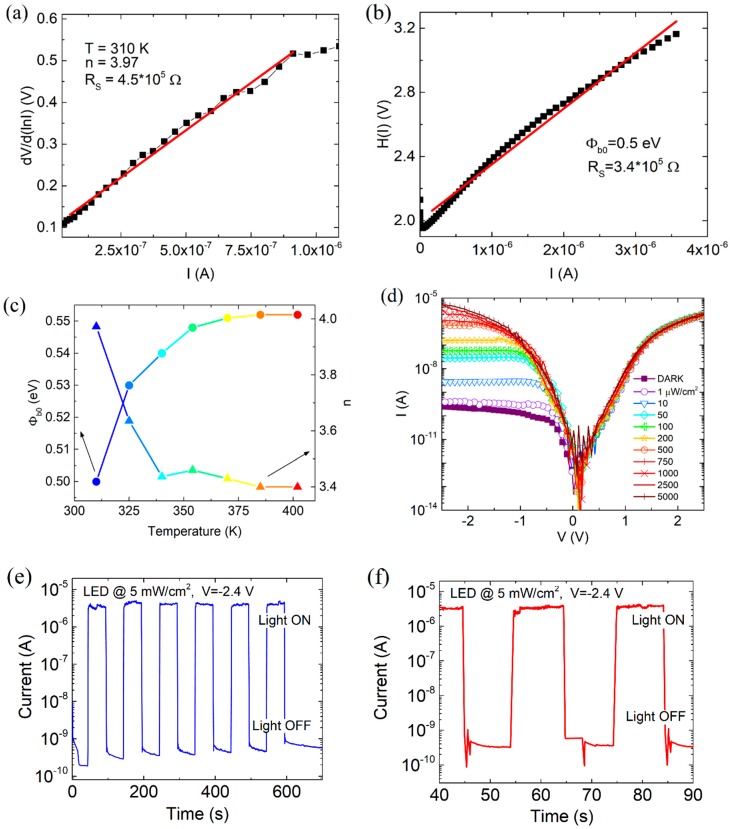
Cheung’s plot of (**a**) d*V*/dln(*I*) versus *I* and (**b**) *H*(*I*) vs. *I*. (**c**) Temperature dependence of ideality factor and barrier height. (**d**) Room-temperature I-V characteristics of the Gr/Si junction under different illumination levels from a white LED array. (**e**,**f**) Dynamic measurement of the photocurrent at reverse bias V=−2.4 V and light intensity of $5\;\mathrm{mW}/{\mathrm{cm}}^{2}$, showing a time response with rise and fall time of ~0.5 s, corresponding to the time resolution of the source-measurement unit.

Figure 2a plots dV/dlnI vs. I at 310 K. In the $R_{S}$ limited region, Equation (7) predicts a straight line with slope and intercept that can be used to extract the series resistance $R_{S}$ and the ideality factor n, respectively.

Once the ideality factor is determined, the right-hand side of Equation (8) can be computed from the current and bias voltage measured in the $R_{S}$ limited region. According to Equation (9), the H(I) vs. *I* plot (Figure 2b), is a straight line whose slope and intercept are the series resistance and the product $n\Phi_{b0}$, respectively. Averaging the values extracted from Equations (7)–(9), $R_{S}∼400\;k\Omega$, while from Equation (9), $\Phi_{b0}\approx 0.52\;\mathrm{eV}$, a value in agreement with the barrier height previously estimated with the Richardson method.

Figure 2c plots the Schottky barrier height and the ideality factor as a function of temperature. The Schottky barrier height $\Phi_{b0}$ increases with the temperature while the ideality factor n decreases. This behavior confirms that the thermionic emission is the dominant carrier conduction process at high temperature, while at lower temperature, the increase of n indicates the presence of other transport phenomena, such as the generation-recombination of charges in the depletion layer and tunneling through the barrier [16,17].

**Figure 3:**
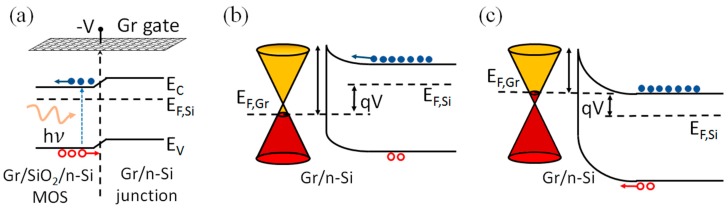
(**a**) Band diagram of the n-type Si substrate along the surface below the Gr/SiO_2_/Si MOS capacitor and the Gr/Si diode, showing that diffusion of photogenerated holes towards the diode area is energetically favored. Band diagram of the Gr/Si junction in (**b**) forward and (**c**) reverse bias (the thin tunneling SiO_2_ interfacial layer is omitted).

We studied the photoresponse of the device by shining light from the top, on the entire device. Figure 2d compares the room-temperature I-V curves obtained in darkness and at different illumination intensities from a white LED lamp. The forward current remains unchanged, while the reverse current increases until it exceeds the forward current at the maximum illumination of $5\;\mathrm{mW}/{\mathrm{cm}}^{2}$. This photocurrent corresponds to a maximum responsivity $ℛ=\left(I_{\mathrm{light}}-I_{\mathrm{dark}}\right)/P_{\mathrm{in}}=2.5\;A/W$, where $P_{\mathrm{in}}$ is the incident optical power, and $I_{\mathrm{light}}$ and $I_{\mathrm{dark}}$ are the reverse currents at V=−2.5 V with and without illumination, respectively. Figure 2e,f displays the reverse current at V=−2.4 V in a sequence of light ON/OFF pulses, and show that the photoresponse of the device is limited by the measurement rate of our source measurement unit (2 Hz). The high photocurrent is the result of the peculiar geometry of the device, which is composed of the Gr/Si diode in parallel with the Gr/SiO_2_/Si MOS capacitor. In forward bias, the positive voltage on graphene causes accumulation of electrons (majority carriers) at the Si/SiO_2_ interface of the MOS capacitor. These electrons do not affect the forward current of the Gr/Si diode, which is controlled by the injection rate of electrons over the barrier. In reverse bias, the electrons cannot overcome the barrier, and the saturation current is mainly due to minority carriers (holes). The reverse current can thus be increased by enhancing the concentration of minority carriers, e.g., by shining light. Upon illumination, photogenerated holes are attracted by the negative bias of graphene to the Si/SiO_2_ interface of the MOS region. Their diffusion to Gr/Si junction originates the observed high photocurrent. Indeed, the motion of such holes to the Gr/Si junction is favored by the band bending from the Gr/SiO_2_/Si to the Gr/Si region, as shown by the band diagrams of Figure 3.

It has been shown that the same mechanism, when applied to thermally generated minority carriers, contributes to the high leakage of the Gr/Si junctions [16].

**Figure 4:**
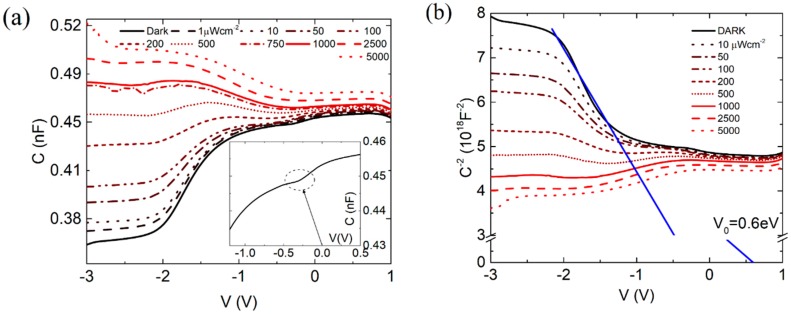
(**a**) Small-signal (100 mV and 10 kHz) C-V measurements in dark and under different illumination levels. The inset highlights a crossover point between two regions where the capacitance of the device is dominated by the Schottky diode (V<−0.25 V) and the MOS capacitor (V>−0.25 V), respectively. (**b**) $\frac{1}{C^{2}}-V$ plot of the device under study.

To further investigate the carrier distribution in the graphene-controlled Si substrate, we performed a room-temperature capacitance-voltage (C-V) characterization in the dark and under illumination (Figure 4). The capacitance measurements were performed in the −3 V to 1 V dc bias range and with ac small-signal of 100 mV amplitude and 10 kHz frequency.

Focusing on the dark curve (black solid line) in Figure 4a, it can be noticed that the measured capacitance is dominated by the MOS capacitor, which is in accumulation, at positive bias. As the voltage is lowered below zero, the MOS enters the weak depletion region, which corresponds to a decreasing capacitance. However, a change in the capacitance behavior occurs at V≈−0.25 V, as highlighted in the inset of the figure. The decreasing capacitance in the range −2 V<V<−0.25 V is likely dominated by the depletion capacitance of the reverse biased Schottky diode. For V<−2 V, the quasi bias-independent capacitance indicates that the MOS, which is now in deep inversion, dominates again over the Schottky diode. Below −2 V, the minority carriers are not able to follow the 10 kHz oscillations, and the MOS is in deep depletion, with the capacitance at its lowest plateau value (the reverse-bias decreasing capacitance of the Schottky diode is negligible in this region). Under high illumination, electron-hole pairs are photogenerated in the depletion region and holes accumulate at the Si/SiO_2_ interface forming an inversion channel, which raises the MOS capacitance to a value comparable to the one in accumulation at positive voltage. Otherwise stated, at the lowest biases and under high illumination, the device shows the typical C-V plots of a MOS on p-type semiconductor. This indicates that the photogeneration rate is able to provide enough positive charge to invert the n-Si and that the channel can follow the oscillation of the small ac signal.

The holes of the inversion channel of the MOS capacitor, injected in the Gr/Si region, cause the high photocurrent of the device, as previously stated.

The interpretation of the Gr/Si dominating capacitance in the range −2 V<V<−0.25 V is confirmed by the linear behavior of the $1/C^{2}$ vs. V plot, shown in Figure 4b. For a Schottky non-ideal diode the inverse square of reverse-bias capacitance is a linear function of the bias [16]:

(10) $$\frac{1}{C^{2}}=\frac{2n\left[n\left(\Phi_{b0}-\Phi_{n}-kT\right)-qV\right]}{A^{2}q^{2}\varepsilon_{S}N_{d}},$$

where $N_{d}$ is the doping density, $\varepsilon_{S}=11.7\varepsilon_{0}$ is the silicon permittivity, and $\Phi_{n}=kT\ln N_{c}/N_{d}$ with $N_{c}=12{\left(2\pi m^{*}\;kTh^{-2}\right)}^{\frac{3}{2}}$, the effective density of states in the conduction band. According to Equation (10), the barrier height $\Phi_{b0}$ can be evaluated from the x-intercept, $V_{0}$, of the straight line fitting the $1/C^{2}$ vs. V plot and results

(11) $$\Phi_{b0}=\frac{V_{0}}{n}+kT\ln\left(\frac{N_{c}}{N_{d}}\right)+kT\approx 0.47\mathrm{eV},$$

($N_{d}=4.5\times 10^{14}{\;\mathrm{cm}}^{-3}$ is the substrate doping and n=3.8 is the measured room-temperature ideality factor). The obtained value of $\Phi_{b0}$ is close to the barrier height extracted from the Richardson’s plot and from the Cheung’s method.

A similar model has been recently proposed in [18], where photocharge generation in the Gr/SiO_2_/Si MOS region has been confirmed by scanning photocurrent measurements. High responsivity, broadband and fast Gr/Si photodetectors were also reported in [19,20].

## 3. Materials and Methods

The device was fabricated from a lightly doped n-silicon wafer with a resistivity of 10 Ωcm. A SiO_2_ layer with a thickness of ∼245 nm was deposited by chemical vapor deposition (CVD), after that a 10 μm-wide trench was patterned by lithography. The SiO_2_ layer was removed from the trench area by a hydrofluoric acid treatment immediately before graphene deposition. This procedure reduced the possibility that oxide will form on the surface. The graphene sheet had been obtained by CVD on Cu foil. In the next step, a graphene sheet of $∼1\times 0.4{\;\mathrm{cm}}^{2}$ was deposited on the wafer, acting both as anode of the Gr/Si junction and top electrode of the Gr/SiO_2_/Si MOS capacitor. Ohmic metal contact to graphene was fabricated by evaporating a Ti/Au metal stack through a shadow mask, while the Si substrate was contacted by depositing Ag paste on an area with removed SiO_2_ and appropriately scratched to produce ohmic contact. For I-V and C-V measurements, the Ti/Au pad was the forcing lead (anode) while the Ag contact on the Si substrate was grounded. Further information about the fabrication process can be found in [16]. Both the I-V and C-V characterizations were performed with the two-probe method, using a Keithley 4200-SCS and a Janis ST-500 micromanipulated probe station. The photoresponse was investigated using a light source consisting of an array of white LEDs with controllable intensity up to $5\;\mathrm{mW}/{\mathrm{cm}}^{2}$ and spectrum in the range 420−720 nm with two peaks at 454 nm and 536 nm, respectively.

## 4. Conclusions

In conclusion, we characterized a hybrid device consisting of a Gr/Si junction in parallel with a Gr/SiO_2_/Si MOS capacitor. We used I-V curves at different temperatures to extract the relevant parameters of the Schottky junction. More importantly, we reported a very high photocurrent, which can exceed the forward current. Using C-V characterization, we proved that the MOS capacitor acts as a reservoir of photo-generated minority carriers. Such excess minority carrier injected into the junction region is the origin of the observed high reverse photocurrent. Hence, the parasitic MOS capacitor enhances the photoresponse of the Gr/Si diode.
